# Heterogeneous monotonic and non-monotonic responses to odor in mitral/tufted glomeruli of the mouse olfactory bulb

**DOI:** 10.1101/2025.02.28.640652

**Published:** 2025-03-06

**Authors:** Narayan Subramanian, David Wharton, Bhargav Karamched, Richard Bertram, Douglas A. Storace

**Affiliations:** 1 Department of Biological Science, Florida State University, Tallahassee, FL; 2 Program in Neuroscience, Florida State University, Tallahassee, FL; 3 Institute of Molecular Biophysics, Florida State University, Tallahassee, FL; 4 Department of Mathematics, Florida State University, Tallahassee, FL

## Abstract

Current models of olfactory sensory processing in the olfactory bulb (OB) posit that both intra- and interglomerular inhibitory circuits are involved in transforming sensory input. However, the impact of these circuits on different olfactory receptor neuron (ORNs) inputs remains poorly understood. We generated a model of the OB input-output transformation in which the output of each glomerulus is a function of its ORN input, local feed-forward intraglomerular inhibition and interglomerular normalization in which activity of each glomerulus is divided by the population response. The output of the model included linear and non-linear concentration-response relationships that depended on the input ORN Hill coefficient and half-activation value. The concentration-response relationships could be broadly categorized into four groups based on how the output response was influenced by increasing the concentration. Increasing concentration evoked monotonic increases (I) or decreases (D) in some glomeruli. Other glomeruli responded with non-monotonic decreases then increases (DI) or increased then decreased (ID). The non-monotonic ID glomeruli required interglomerular inhibition in our model, were most common in glomeruli with higher affinity ORN input and were heterogeneous in the magnitude of their drop. *In vivo* 2-photon Ca^2+^ imaging from MTC glomeruli in awake mice revealed qualitatively similar response types. Increasing levels of excitation drove higher levels of suppression in subsets of glomeruli, and nearly half of the recorded MTC glomeruli could be classified as ID. Additionally, the sensitivity of individual glomeruli was significantly correlated with the degree to which it was non-monotonic. Our results demonstrate that nonlinear responses of MTC to changes in odor concentration are not unusual, but indeed are typical, and that they can be explained by intra- and interglomerular inhibition.

## Introduction.

One of the goals of neuroscience is to define the underlying neural circuits and computations involved in transforming sensory signals into behaviorally relevant information (Carandini, 2012; Kanter et al., 2022; Langdon et al., 2023). For example, odor recognition must be initiated by the binding of an odor ligand to olfactory receptor neurons (ORNs), each of which express one kind of olfactory receptor (OR) protein (out of ~1000) and have a distinct affinity for each odor (Buck and Axel, 1991; Malnic et al., 1999; Reisert and Matthews, 1999; Araneda et al., 2000; Xu et al., 2020; Zak et al., 2020). Odors evoke varying degrees of activity across the receptor population that must be related to perception. However, low concentrations of an odor will activate high affinity receptors, while higher concentrations of the same odor will saturate those high affinity receptors and recruit low affinity receptor types (Wachowiak and Cohen, 2001; Storace and Cohen, 2017; Storace et al., 2019; Hu et al., 2020). Consequently, there is a confound between how the identity and concentration of an odor are encoded at the level of ORNs. Without further processing odors would be perceived differently when they are experienced at different concentrations or in the presence of other background odor stimuli.

Each ORN type maps to the olfactory bulb (OB) in OR specific channels called glomeruli (Buck and Axel, 1991; Malnic et al., 1999). Mitral/tufted cells (MTCs) extend their apical dendrite into a single glomerulus and project their axons to the rest of the brain (Nagayama et al., 2010; Igarashi et al., 2012). Therefore, each glomerulus contains the input-output transformation for a given olfactory receptor, which can be influenced by a complex synaptic network that includes inhibitory interneurons (Cleland and Sethupathy, 2006; Parrish-Aungst et al., 2007; Cleland, 2010; Nagayama et al., 2014). The inhibition provided by interneurons is an important element of the neural processing of ORN input and comes in two forms. One involves feed-forward inhibition where high-sensitivity inhibitory interneurons are excited by ORN input and actively inhibit MTCs (Cleland and Sethupathy, 2006; Gire and Schoppa, 2009; Shao et al., 2009). A second process involves normalization in which MTC output reflects a combination of its afferent input, as well as the collective activity across a population (Cleland et al., 2007; Carandini and Heeger, 2011). Both processes are thought to be important in performing sensory transformations that may be important in expanding the dynamic range of ORN inputs, enhancing contrast across glomeruli, decorrelation and normalization (Cleland, 2010; McGann, 2013; Martelli and Storace, 2021).

The goal of this study was to begin to understand how these two forms of inhibition can transform different ORN inputs across concentration changes. We implemented a mathematical model in which the input from different ORN types were transformed by intraglomerular feed-forward inhibition and interglomerular normalization. Our model produced a heterogeneous mixture of linear and nonlinear output response types that depended on the ORN Hill coefficient and its half-activation value. The glomerular output could be broadly categorized into four groups based on how the concentration-response relationship was influenced by increasing concentration. Some glomeruli exhibited monotonically increasing (I) or decreasing (D) relationships. Other glomeruli exhibited non-monotonic relationships that included those that transitioned from suppression to excitation (decreasing-increasing, DI) or instead increased to a point at which further concentration increases evoked smaller responses (increasing-decreasing, ID). Importantly, these different response types were present within the same concentration range. The ID response type required the presence of increasing levels of inhibition within the glomerular circuit and typically originated from higher affinity ORN inputs.

We used 2-photon Ca^2+^ imaging in awake mice to test whether MTC glomerular responses measured across a large concentration range exhibited similar response categories. Within the same imaging field of view, MTC glomeruli responded to odors with linear and non-linear concentration-response relationships that closely approximated the kinds of responses predicted by the model in an odor-specific manner. Additionally, we found a significant positive correlation between the levels of excitation and inhibition across the MTC glomerular population. Nearly half of the glomerular population responded with ID concentration-response types, and the sensitivity of each glomerulus was predictive of the magnitude of the non-monotonic component.

Together the results support a model in which the OB input-output transformation of each glomerulus is shaped by the balance of its input excitation and rising inhibition. Increases in concentration will evoke higher levels of excitation across the glomerular population, which causes an increase in the magnitude of inhibition. High affinity olfactory receptor types will saturate in response to higher concentrations and be susceptible to the influence of local and lateral inhibitory processes. We propose that non-linear MTC concentration-response relationships are the natural consequence of ORNs with a small dynamic range and intra- and interglomerular inhibition.

## Methods.

A subset of these data were included in a previous publication that analyzed different properties of MTC odor responses (Subramanian et al., 2025).

### Mathematical Modeling.

The model is based on ideas described in (Cleland and Sethupathy, 2006). The output of the ORNs is dependent upon the odorant concentration, [odorant], and is described mathematically by the sigmoidal Hill function:

(1) $$\text{ORN=}\frac{v{\left[\text{odorant}\right]}^{n}}{\kappa^{n}+{\left[\text{odorant}\right]}^{n}}$$

All variables and parameters are dimensionless, and [odorant] ranges from 0 to 1. The parameter v sets the maximum value of ORN and is assumed to be the same for all ORNs. The other two parameters are the half-activation constant, κ, and the Hill coefficient, n, which sets the steepness of the ORN’s response (higher values of *n* yield steeper response functions). To generate a large (*N* = 949) heterogeneous population of ORNs, *N* combinations of parameter values were sampled from a uniform distribution on the intervals of (0,2) for κ and (1,4) for n. The ORN signal is then subject to presynaptic inhibition from other glomeruli before entering the target glomerulus, producing a “normalized ORN” value $\bar{ORN}$:

(2) $$\bar{\text{ORN}}=\frac{\text{ORN}}{1+\mu }$$

where $\mu =\sum_{i=1}^{N}$ ${\text{ORN}}_{i}/N$ is the average value of all ORN activity. This normalized output of the ORN is then the input to periglomerular and MTCs, reflected in the variables PG and MTI, respectively. These are also described with increasing Hill functions:

(3) $$\text{PG=}\frac{\beta {\bar{\text{ORN}}}^{4.5}}{\kappa_{p}^{4.5}+{\bar{\text{ORN}}}^{4.5}}$$

(4) $$\text{MTI=}\frac{{\bar{\text{ORN}}}^{3}}{\kappa_{m}^{3}+{\bar{\text{ORN}}}^{3}}$$

where β, $\kappa_{p}$, and $\kappa_{m}$ are parameters that are assumed to be the same for each PG and MTI. They are set so that PG begins to activate at lower values of $\bar{\text{ORN}}$ than MTI and saturates at a lower value (saturation value of β) than MTI (saturation value of 1). The output of each glomerulus, MTC output, is then the difference between MTI and the inhibition from PG cells,

(5) MTCoutput=MTI−PG

Parameter values are given in Table 1.

### Transgenic mice.

GCaMP6f was targeted to MTC glomeruli by mating the Ai148 GCaMP6f transgenic reporter line (Jax stock #030328) to the Tbx21-cre transgenic line (Jax stock #024507) (Mitsui et al., 2011; Daigle et al., 2018; Subramanian et al., 2025). Genotyping was performed by Transnetyx (Cordova, TN) and offspring that expressed eGFP and Cre recombinase were used for experiments. Appropriate targeting of GCaMP to MTC glomeruli was confirmed histologically based on endogenous fluorescence expression.

### Surgical procedures.

All procedures were approved by the Florida State University Animal Care and Use Committee. Male and female adult (> 21 days) transgenic mice were anesthetized using ketamine/xylazine (90 / 10 mg/kg, Zoetis, Kalamazoo, MI), placed on a heating pad and had ophthalmic ointment applied to their eyes. Mice were given a pre-operative dose of carprofen (20 mg/kg, Zoetis, Kalamazoo, MI), atropine (0.2 mg/kg, Covetrus, Dublin, OH), dexamethasone (4 mg/kg, Bimeda, La Sueur, MN), and bupivacaine (1.5 mg/kg, Hospira, Lake Forest, IL). Fur was removed from the top of the skull using a depilatory agent and rinsed, after which the skin was scrubbed with 70% isopropyl alcohol and iodine (Covidien, Mansfield, MA). An incision was made to remove the skin over the skull and blunt dissection was used to remove the underlying membrane. Dental cement (Metabond, Covetrus, Dublin, OH) was used to attach a custom headpost to the skull, which was held using a custom headpost holder. After the cement finished drying, the bone above the OB was either thinned using a dental drill (Osada, XL-230, Los Angeles, CA) and covered with cyanoacrylate to improve optical clarity or was removed and replaced with #1 cover glass. Upon completion of the surgery, the mouse was allowed to recover on a heating pad until they were fully ambulatory. Animals were given a post-operative dose of carprofen at the end of the day of surgery and for at least 3 days post-operatively.

### Histology.

Mice were euthanized (euthasol) following imaging and either underwent cardiac perfusion with phosphate buffered saline and 4% paraformaldehyde or had their brains directly extracted and post-fixed in 4% paraformaldehyde before being cut on a vibratome in 40 µm sections (Leica VT1000S, Deer Park, IL). OB sections were mounted on slides and were coverslipped using Fluoromount-G containing DAPI (SouthernBiotech, Birmingham, AL). Endogenous fluorescence expression of GCaMP6f was observed using a GFP filter set on either a Zeiss Axioskop epifluorescence microscope or a Nikon CSU-W1 spinning disk confocal microscope.

### 2-photon imaging.

Imaging was performed using a Sutter MOM 2-photon microscope equipped with an 8 kHz (30.9 Hz) resonant scanner (Cambridge Technology, USA) and an emission pathway equipped with a GaAsP PMT (#H10770PA-40–04, Hamamatsu, Japan). Laser excitation was provided using a Spectra-Physics DS+ between 940–980 nm with power modulated by a Pockels cell (Model #350–105-02, Conoptics, Danbury, CT). Imaging was performed using a Nikon 16× 0.8 N.A. objective lens. Laser power was confirmed to be less than 150 mW at the output of the objective lens measured using a power meter (Newport 843-R) for scanning areas ranging between 711 µm^2^ - 1138 µm^2^.

### Odorant delivery.

Methyl valerate (CAS #624–24-8), isoamyl acetate (CAS #123–92-2), benzaldehyde (CAS #100–52-7), acetophenone (CAS #100–52-7), and 2-phenylethanol (CAS #60–12-8) (Sigma-Aldrich, USA) were used at concentrations between 0.05 and 5.5 % of saturated vapor. The olfactometer design involved air being pushed through vials of pure odor using a syringe pump (NE-1000, PumpSystems, Farmingdale, NY) running at different flow rates (0.25 – 28 ml /min). This odor stream underwent an initial air dilution with a lower flow rate of clean air (30 ml/min). The resulting odorized air stream connected to a dual 3-way solenoid valve (360T041, NResearch, West Caldwell, NJ) which was connected to an exhaust, a clean air stream and a Teflon delivery manifold which served as the final delivery apparatus placed in front of the mouse’s nose. The delivery manifold had a higher flow rate of clean air constantly flowing through it (450 ml/min), providing a second air dilution. Prior to odor triggering, the solenoid sent the odorized air stream to the exhaust, and the clean air stream to the animal. Triggering the solenoid caused the odor to be injected into the delivery manifold. The odor delivery time-course was confirmed using a photoionization detector (200C, Aurora Scientific, Aurora, ON) (Storace and Cohen, 2017, 2021; Subramanian et al., 2025).

### Imaging procedures.

Prior to data collection mice were positioned underneath the microscope and the headpost holder angle was adjusted to optimize the imaging field of view. During data collection, awake head-fixed mice were placed underneath the microscope objective with the olfactometer and a thermocouple (to measure respiration, Omega 5TC-TT-K-36–36, Newark) near its nose. The signals from the respiration sensor were amplified and low-pass filtered using a differential amplifier (Model 3000, AM-Systems, Sequim, WA), which was recorded by the imaging system. Odors were delivered at concentrations between 0.05 – 5.5 % of saturated vapor in trials separated by a minimum of 3 minutes. For each animal, we prioritized measuring multiple repetitions for each concentration for a particular odor before a second odor was attempted.

### Data analysis.

#### Frame Subtraction Analysis.

The mean fluorescence and frame subtraction images are from the average of at least two single trials (Figure 2). The mean fluorescence images are generated from the average of all the frames during the imaging trial. The frame subtraction images were generated by subtracting an average of the 19 frames during odor stimulation from the average of 9 frames prior to the stimulus. The resulting image underwent two passes of a low-pass spatial filter and the fluorescence (F) values were converted to ΔF/F by dividing the fluorescence value of each pixel by the mean of at least 60 consecutive frames in the image stack in Turbo-SM. The intensity scale range is fixed to the same minimum and maximum range for all concentrations for each odor (Figure 2).

#### Processing and segmentation.

Following data acquisition, the raw image files were spatially and temporally averaged from 512×512 pixels sampled at 30.9 Hz to 256×256 pixels sampled at 7.72 Hz. The resulting data were exported to TIFF format for all subsequent analysis. Occasional recordings with motion artifact that made it impossible to interpret the measurements were discarded from subsequent analysis. Glomerular regions of interest were manually segmented in custom software (Turbo-SM, SciMeasure, Decatur, GA) and were identified based on their morphological properties in the mean fluorescence, and functional responses in a frame subtraction. The pixel areas containing the regions of interest were saved and the fluorescence time course values from each region of interest were extracted for subsequent analysis. Fluorescence time course values were converted to ΔF/F by dividing each trace by the mean of the frames prior to odor stimulation. Odor response amplitudes and corresponding Z-Scores were calculated as the largest difference between a 1200 msec window during the odor presentation and the time prior to odor stimulation (e.g., Figure 3B-C).

#### Other analyses.

Population descriptive statistics were quantified in responsive glomeruli which were defined as those in which an odor evoked a minimum of a 5 standard deviation change from baseline at any concentration (Figure 3E). Threshold and best response are defined as the lowest concentration evoking a response, and the concentration evoking the largest response, respectively (Figure 3F-G). For all other analyses, glomeruli were included that responded with a minimum of 3 standard deviations above baseline at some concentration.

Population measurements of the different response categories were generated by averaging the fluorescence time course from all the glomeruli in a field of view assigned to that category (Figure 4E). The relationship between excitation and suppression was quantified by averaging the response from all glomeruli that responded to the highest concentration with excitation and suppression, respectively (Figure 5). The mean excited and suppressed response for each preparation are plotted together, with the responses from individual preparations connected with a line (Figure 5E). The correlation between these values were quantified for individual preparations and across the population using the corrcoef function in MATLAB (Figure 5D-E). The variance explained was quantified by binning the mean excitation and suppression value measured for each concentration (Figure 5E, black solid line), and fitting them with a sigmoid (Figure 5E, black dashed line).

The Monotonicity Index (MI) is a metric that computes the degree of non-monotonicity in a concentration-response function (Escabí et al., 2007; Higgins et al., 2008). The MI of individual glomeruli was calculated by computing the d-prime value by measuring the concentration-response function (CRF) in ΔF/F.


(6) $$D^{\prime }=\frac{CRF=CRF_{max}}{\sqrt{\sigma_{max\;}^{2}+\sigma^{2}}}$$

The ${\text{CRF}}_{\max}$ is the response at the concentration with the largest ΔF/F change from the baseline and CRF is the ΔF/F change at each concentration. $\sigma_{\text{max}\;}^{2}$ is the response variance at the highest odor concentration, while $\sigma^{2}$ is the response variance at each odor concentration. MI was then computed as the minimum D’ measured at concentrations beyond the one evoking the maximum ΔF/F response. Consequently, a monotonically increasing glomerulus is assigned a value of 0, while glomeruli with non-monotonic concentration response functions are assigned negative values. Glomeruli with moderate non-monotonic shapes are closer to 0, while those with a larger decrease are assigned increasingly negative values. MI values were quantified for all glomeruli in the data set (independent of the categories described in Figure 4), except for exclusively suppressed glomeruli because they are assigned large MI values (Figure 6).

Half-maximum values were quantified by performing a linear interpolation of the concentration-response relationship for each glomerulus (linearinterp fittype in MATLAB). The concentration evoking the half-maximum response in the linear interpolation was selected with one caveat. Glomeruli with a response to the lowest concentration that was greater than the half-maximum were assigned a half-maximum value of 0.05% (e.g., Figure 6B, roi9).

Hill equation fits of monotonically increasing glomeruli were performed in glomeruli exhibiting MI values greater than −0.5, Hill fits were calculated using the following equation where X is the response (in ΔF/F) of the glomerulus at each concentration, and k is the half-saturating value estimated from a linear interpolation of the concentration-response function.


(7) $$\frac{X^{n}}{X^{n}+k^{n}}$$

Fits were only analyzed if they met the following criteria: the Hill coefficient was between 0.75 and 6; the r^2^ value was greater than 0.9, the root mean sum of squares was less than 0.1 and the sum of square error was less than 0.1 (Zak et al., 2020).

For the spatial maps of glomerular ΔF/F and categorical responses, glomeruli that did not respond at any concentration are indicated using cross-hatching (Figure 7A-B). For responsive glomeruli, concentrations that evoked a change of less than 2 standard deviations were assigned a value of zero to facilitate visual analysis (Figure 7A, *white polygons*).

## Results

### A model that combines intra- and interglomerular processing predicts four types of concentration-response relationships in MTC glomeruli.

**Figure 1:**
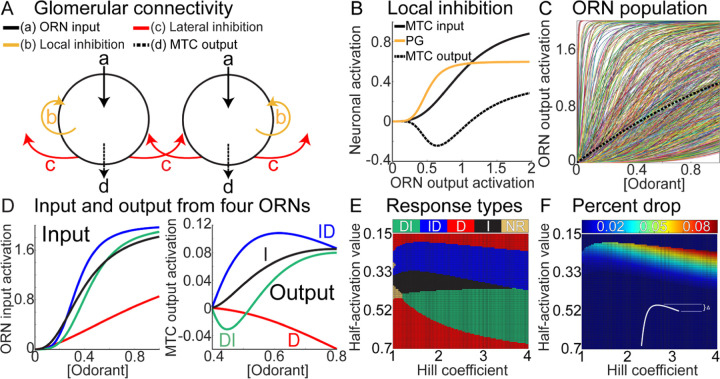
(A) Model of the glomerular input-output transformation. (B) Local inhibition alone generates a half-hat response in MTCs. (C) The population of ORNs in the model (thin lines) used to calculate the normalization function to perform lateral presynaptic inhibition (black dashed line). (D) The concentration-response relationships of four ORNs (left) and their corresponding MTC output reflecting both local and lateral inhibition (right). Output responses were categorized based on the shape of the concentration-response relationship: I, increasing; ID, increasing then decreasing; DI, decreasing then increasing; D, decreasing. (E) MTC responses for different ORN Hill coefficients and half-activation values. (F) The percent decrease from the maximum value for combinations of Hill coefficients and half-activation values that result in an ID response.

We generated a model to better understand how intra- and interglomerular processing transforms the concentration-response properties of different ORN inputs (see Methods for model equations and parameter values). The output of each glomerulus (Figure 1A, d) was a function of its ORN input (Figure 1A, a), input from local PG interneurons that are activated by ORN input and provide inhibition onto the MTC output (Figure 1A, b), and inhibitory interglomerular connectivity acting presynaptically to scale down the ORN input (Figure 1A, c).

ORNs modeled using the Hill equation served as input both to MTCs and inhibitory PG interneurons whose activation was similarly described by a Hill function, but with the PG response function left-shifted relative to the MTC response function (Figure 1B, PG). The difference between these two response functions was then taken as the output from the MTC (Figure 1B, MTC output). The MTC output, which is relative to the basal level of activity, has a half-hat shape that shows a dip to negative values for low ORN activation and an increase for higher ORN activation (Figure 1B, MTC output).

Interglomerular processing was modeled by generating a population of 949 ORNs with Hill coefficients sampled from a uniform distribution from 1 to 4, consistent with previously published work (Figure 1C, thin lines) (Firestein and Zufall, 1993; Wachowiak and Cohen, 2001; Storace and Cohen, 2017; Zak et al., 2020; Platisa et al., 2022). Odor affinities (their half-maximum values) were sampled from a uniform distribution on the interval between 0 and 2 (Figure 1C). The model is dimensionless, as are the half-maximum values for the receptors. The ORN input to the MTCs and inhibitory PG cells were divided by the mean of the ORN activation function to reflect lateral presynaptic inhibition from all glomeruli (Figure 1C, dashed black line).

Four examples of ORN input and corresponding MTC output across a concentration range are illustrated in Figure 1D (the colors are matched across the two subpanels). The model output in response to four input functions could be broadly described as having concentration-response relationships that monotonically increased (I), monotonically decreased (D), transitioned from suppressed to excited (DI) or increased to a point at which further concentration increases evoked progressively smaller responses (ID) (Figure 1D, right subpanel).

The types of MTC responses are shown with color coding in Figure 1E over a grid of values of the ORN Hill coefficient and half-activation constants. The ORNs with the highest affinities had a purely decreasing response to increasing odor concentrations (Figure 1E, D, top red regions). In these cases, the MTC output reached its maximum at low odor concentrations and could only decline due to lateral inhibition as other glomeruli became activated at higher concentrations. For ORNs with lower, but still relatively high affinities, the typical response was an initial increase followed by a decrease (Figure 1E, ID, blue region). In these cases, strongly activated ORNs near saturation were dominated by increasing lateral inhibition. For lower-affinity ORNs the response was purely increasing, particularly when the ORN response function was relatively linear (Figure 1E, I, black region). At even lower affinities the typical response was a decrease, followed by an increase (Figure 1E, DI, green region). In this case, inhibition dominated the MTC output prior to its strong activation at higher odor concentrations, producing a DI response. Finally, at the lowest affinities the response was purely decreasing, reflecting dominance of inhibition over all odor concentrations considered (Figure 1E, D, red region).

In principle, D responses can be generated by feed-forward suppression within a range of concentrations (e.g., Figure 1C, dashed line between 0 and 0.6), or in the case of spontaneously active ORNs suppressed by interglomerular inhibition. ID responses cannot, however, result from the local inhibition motif used in our model. Instead, they depend upon lateral inhibition, which we hypothesize results from higher levels of interglomerular inhibition resulting from activation of lower affinity ORN glomeruli (Figure 1C, black line). A consequence of this framework is that the magnitude of non-monotonicity should be heterogeneous and originate from higher affinity glomerular input that saturates at lower concentrations. A quantification of the percent decrease from the maximum over the grid of ORN parameters evoking ID responses revealed a gradient of non-monotonic drops (Figure 1F). Most of the parameter values in the grid that produced a large drop in MTC activation following a peak had small ORN half-activation values indicating high affinity for the odor.

The structure of the interglomerular inhibitory network is currently unknown (Fantana et al., 2008; Banerjee et al., 2015; Economo et al., 2016). The results shown in Figure 1 were obtained using all-to-all coupling (a clique) among the glomeruli. We also considered the case in which input from 10 randomly chosen glomeruli provided the interglomerular inhibition, which resulted in a different average ORN activation curve which was used in the normalization of ORN input to glomeruli (Eq. 2) (*not shown*). Generally similar response categories were present when performing normalization based on this random subset of ORNs. Most importantly, all four types of response patterns were produced, though the grid of ORN parameters that produced the different types of patterns was changed (not shown). It is clear, then, that the four different response types are generic, while the shapes and extent of the regions in the 2-parameter grid that produce the different types of responses depend on the structure of the interglomerular inhibitory network.

### In vivo measurements of concentration-response relationships in MTC glomeruli.

**Figure 2:**
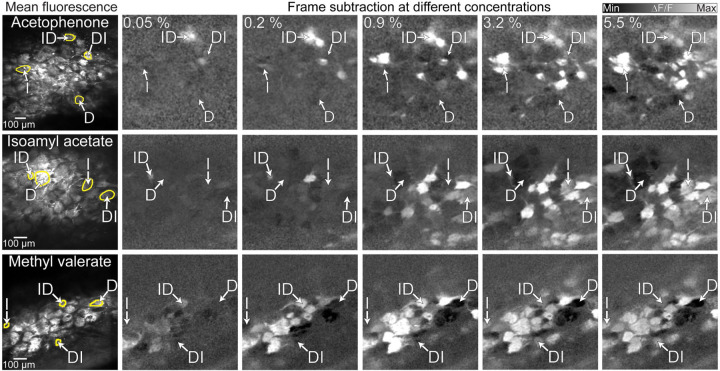
Mean fluorescence (left subpanel) and frame subtraction analysis illustrating MTC activation at different concentrations in three preparations (rows). The intensity scale is fixed across all concentrations for each preparation. The arrows highlight glomeruli with increasing (I), decreasing (D), increasing-decreasing (ID), and decreasing-increasing (DI) concentration-response relationships.

We tested these predictions in a data set in which odor responses were measured from MTC glomeruli across a ~100-fold change in concentration using *in vivo* 2-photon Ca^2+^ imaging in awake mice. Imaging was performed in transgenic mice in which GCaMP6f was selectively expressed in MTCs and their corresponding apical dendrites innervating the glomerular layer (Figure 2, *mean fluorescence*) (Subramanian et al., 2025). Frame subtraction analyses from different preparations illustrate that odors evoked different patterns of excited and suppressed responses within the same field of view (Figure 2, *white versus black regions in frame subtractions*). The specific activation pattern across the glomerular population was a complex function of odor concentration (Figure 2, *compare over concentration range 0.05 to 5.5%*). Many glomeruli became increasingly excited (monotonically increasing, I) or suppressed (monotonically decreasing, D) at higher concentrations (Figure 2, I vs D). Some glomeruli exhibited nonlinear changes in response to increasing concentration in which they first decreased then increased (DI), while others increased and then decreased (ID) (Figure 2, DI vs ID).

**Figure 3:**
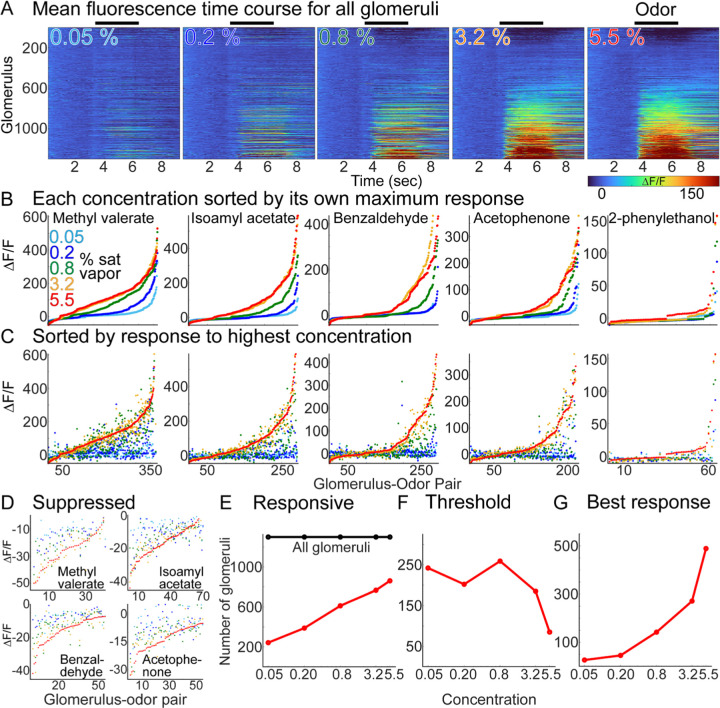
(**A**) Fluorescence time course from all glomerulus-odor pairings. The color scale is fixed across the 5 concentrations. (**B**) Response to all glomeruli for each odor sorted by the maximum response to each concentration (colors). (**C**) Same arrangement as panel **B** except that glomeruli are sorted by the maximum response to the highest concentration. (**D**) Data from panel **C** cropped to visualize suppressed glomeruli. (**E**-**G**) Descriptive statistics.

The response from each glomerulus was segmented, and the fluorescence time course and odor response amplitudes (ΔF/F) and corresponding Z-scores were calculated as the largest difference in a 1200 msec moving window between the odor stimulation period and the baseline fluorescence. We quantified the concentration-response relationships in a population of glomerulus-odor pairings that included 736 individual glomeruli in 20 different preparations (36.8 ± 3.3 glomeruli per preparation, ranging from 13–64). At least one odor was delivered to each preparation at 5 different concentrations, which included the odors methyl valerate (n = 14), isoamyl acetate (n = 7), benzaldehyde (n = 7), acetophenone (n = 5), and 2-phenylethanol (n = 1). The data set includes 6495 glomerulus-odor pairings across all tested odors and concentrations (Figure 3A). The fluorescence time course versus amplitude of all glomerulus-odor pairings illustrates that higher concentrations typically evoked stronger degrees of excitation and suppression in more glomeruli (Figure 3A), though there are many instances where this is not true, as discussed next.

We examined the relationship between concentration and response magnitude at the population level by plotting the ΔF/F evoked for each glomerulus-odor pairing for different odors (Figure 3B-C). Sorting glomerular responses from smallest to largest for individual concentrations illustrates that relatively few glomeruli are activated at low concentrations, while the number of activated glomeruli and their overall response amplitude increased at higher concentrations (Figure 3B). Sorting glomeruli by their response to the highest concentration more clearly visualizes concentration-response heterogeneity across the population (Figure 3C). For many glomeruli, the response to the highest concentration evoked the largest amplitude response (Figure 3C, the red point is above the others), although many responded with a sub-maximal response to the highest concentration (Figure 3C, points of other colors are above red). For glomeruli with suppressed responses, the highest concentration tended to evoke the strongest magnitude of suppression, but not always (Figure 3D, data are cropped from panel C).

We quantified these observations using a statistical threshold in which significantly responsive glomeruli were defined as responding to the odor with a 5 standard deviation change from the pre-odor frames. The number of significantly responsive glomeruli increased as a function of odor concentration (Figure 3E). A similar analysis was used to define the lowest concentration that evoked a significant response for each glomerulus (the “threshold” concentration), and the concentration that evoked the strongest response (the “best” concentration). Of the responsive glomeruli, approximately half had a threshold response of 0.9% of saturated vapor, while the remaining first responded to a lower or higher concentration (Figure 3F). About half of the glomeruli responded most strongly to the highest tested concentration, while the remainder responded mostly strongly to a lower concentration (Figure 3G). Increasing or decreasing the statistical threshold altered the number of “responsive” glomeruli in the data set, but did not substantially change the mean threshold and best response of the glomeruli (*not shown*).

### Examples of different MTC response types.

**Figure 4:**
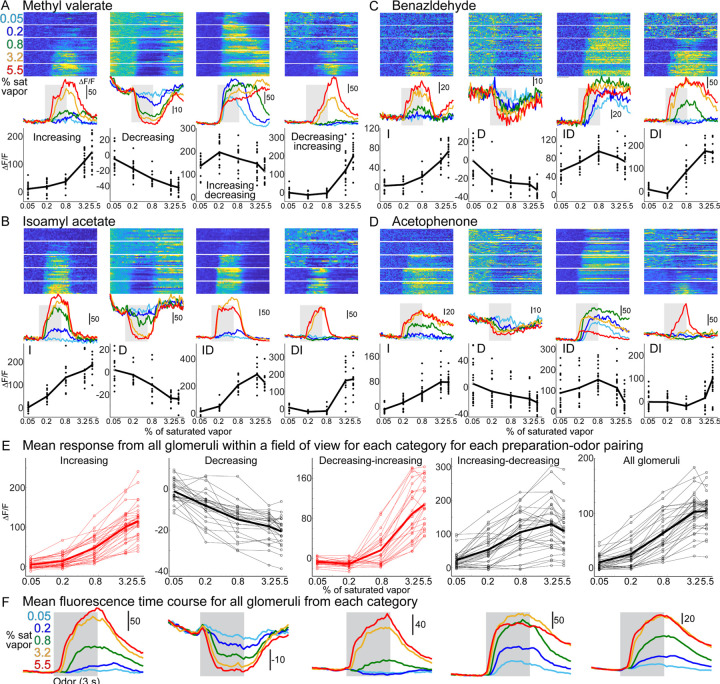
(**A**-**D**) Single trials (top), mean fluorescence time course (middle) and concentration-response relationships with points indicating the response amplitudes from the single trials for four different preparations. (**E**) Mean concentration-response relationships for each preparation-odor pairing for each response type (1st four subpanels), and across all glomeruli in each field of view (right most subpanel). (**F**) Mean fluorescence time course from all glomeruli in each category.

Representative examples from four different preparation-odor pairings illustrate that linear and non-linear glomerular concentration-response relationships were consistent across single trials and often present in the same field of view (Figure 4A-D, all four glomeruli in each subpanel were simultaneously imaged in the same field of view). Glomeruli categorized as I exhibited continuously rising response amplitudes as a function of concentration, while D glomeruli were more strongly suppressed at higher concentrations (Figure 4A-D, 1^st^ two subpanels). Glomeruli consistent with the ID response type were also present where higher concentrations drove increased response amplitudes up to a point at which further increases caused progressively smaller, but still excited responses (Figure 4A-D, 3^rd^ subpanel). The time course and single trial data for non-monotonic glomeruli highlight the stability of these responses and that the non-monotonic decrease is not a rapid adaptation to the stimulus but instead is a smaller response during the odor presentation (Verhagen et al., 2007; Lecoq et al., 2009). Other glomeruli exhibited responses that were characteristic of the DI response type, which were suppressed at a lower concentration, and transitioned to increasingly excited responses at higher concentrations (Figure 4A-D, 4^th^ subpanel).

I and D response types were present in 336 and 125 glomeruli, respectively (Figure 4A-D). I glomeruli could be well fit with the Hill equation with a mean Hill coefficient of 2.7 ± 0.08 (range of 0.8–5.9, included 190 glomeruli in 16 preparation-odor pairings, the remainder were excluded due to poor fits, see methods). ID concentration-response relationships were measured in 362 glomeruli. DI responses were present in 92 glomeruli and by definition were suppressed at some concentration but responded to the highest concentration with an increase in fluorescence. Most DI glomeruli were suppressed at 0.2% (42/92) or 0.9% (35/92) of saturated vapor, respectively. Finally, 34 glomeruli exhibited concentration-response relationships that included suppression but could not be clearly fit into any category (e.g., glomeruli that transitioned from strongly excited to suppressed, *not shown*). Thus, of the 915 glomeruli that could be categorized, 37% were of type I, 14% were of type D, 10% were of type DI, and 39% were of type ID.

Plots in which the concentration-response relationship of all glomeruli from each category were averaged together in each preparation-odor pairing illustrate that each response type was consistently present in nearly all preparations (Figure 4E, left 4 subpanels, each line represents the average from one preparation). Averaging all responsive glomeruli together within the same field of view demonstrates that higher concentrations drives overall increasing levels of excitation across the glomerular population (Figure 4E, All glomeruli, right-most subpanel), although this is often not true at the level of a single glomerulus. The correlation between concentration and mean response was significantly correlated for most individual preparations (0.67 to 0.97, mean of r = 0.87 ± 0.08, p < 0.05 in 18/32 preparation-odor pairings). Therefore, excitation scales with concentration in MTC glomeruli.

### Excitation scales with suppression in MTC glomeruli.

**Figure 5:**
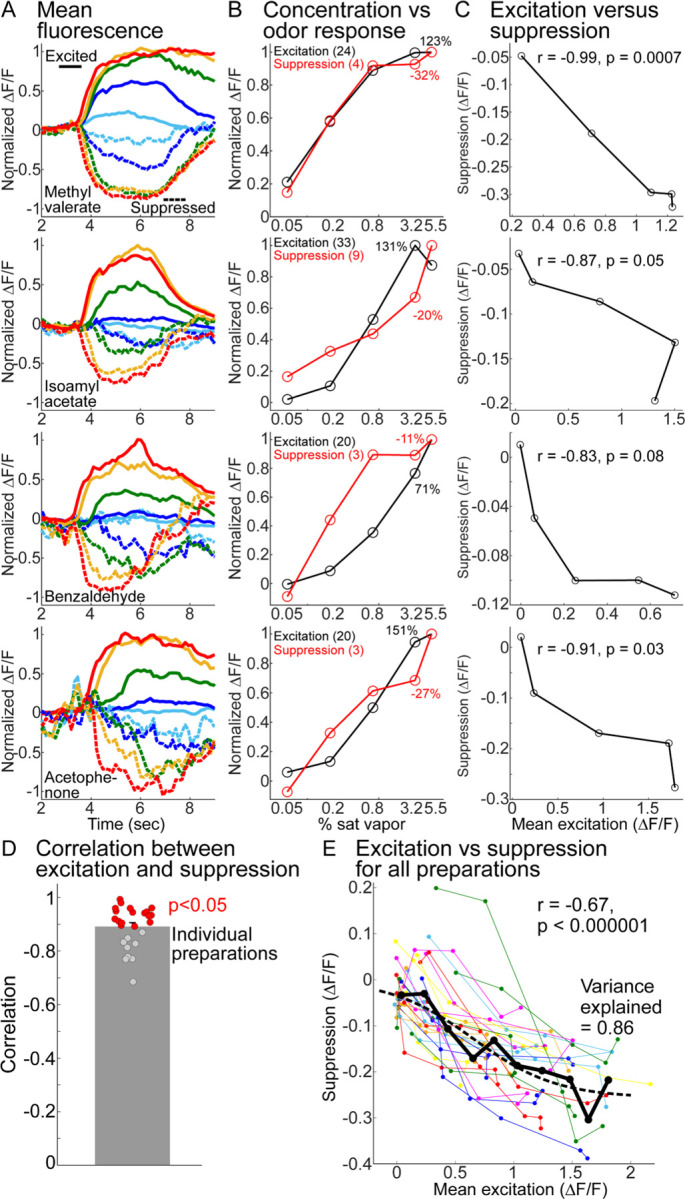
(**A**-**C**) Mean fluorescence time course of excited (solid traces) and suppressed (dashed traces) glomeruli (A), concentration versus normalized ∆F/F (**B**), and excitation versus suppression (**C**) for four preparations. The number of glomeruli averaged together and the maximum ∆F/F values are indicated in subpanel **B**. (**D**) Correlation between excitation and suppression for each preparation. (**E**) Mean excitation vs suppression for all preparations. Each individual point is the average of all the excited and suppressed glomeruli within a field of view for one concentration-odor pairing. Measurements from the same preparation-odor pairing are connected with a line. The individual points were binned (solid black line and black circles) and fit to a sigmoid (dashed line).

Another prediction from our model is that higher levels of excitation should evoke increasing levels of inhibition (Figure 1C, red line). We tested this prediction by comparing the mean of all excited glomeruli within a field of view against the mean of all D glomeruli within a field of view. The response to all concentrations was included for each glomerulus if it responded at any concentration. In four exemplar preparation-odor pairings, there was a strong and sometimes statistically significant relationship between normalized excitation and suppression (Figure 5A-C, the number of excited and suppressed glomeruli in each preparation-odor pairing is indicated in panel B).

The relationship between excitation and suppression was significantly correlated in 18 out of the 27 preparation- odor pairings included in the analysis (Figure 5D, mean r = 0.89 ± 0.01, statistically significant preparations are indicated in red), and when combining all preparation-odor pairings together (Figure 5E, r = −0.67, p < 0.001). Additionally, the individual points from all preparations were binned together, the values of which were fit to a sigmoid (Figure 5E, thick black line and circles and the dashed line; different fitting parameters yielded similar fits). Although there was variance in the mean values, there was a clear sigmoidal decline which accounted for a substantial amount of the variance (Figure 5E). Therefore, suppression scales with increasing levels of excitation in subsets of MTC glomeruli.

### MTC glomeruli ID concentration-response relationships are heterogeneous and have more sensitive odor responses.

**Figure 6:**
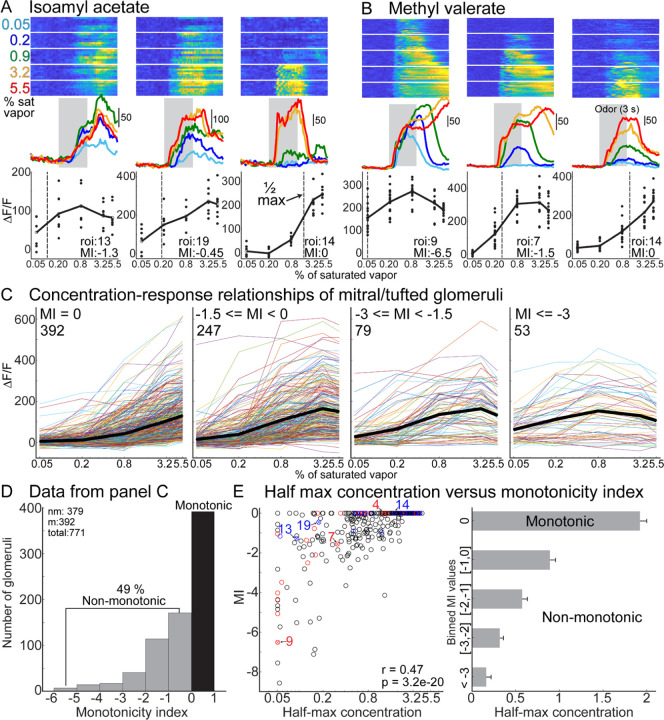
(**A**-**B**) Single trials (top row), mean fluorescence time course (middle) and mean concentration-response relationships (bottom) for three MTC glomeruli in two preparation-odor pairings. The vertical lines in the bottom subpanel of **A**-**B** indicate the half-maximum concentration. (**C**) Concentration response relationships for all responsive MTC glomeruli from all preparations binned by their MI, with the mean indicated with a black line. The number of glomeruli in each bin is in the top left of each panel. (**D**) Histogram of MI values from panel **C**. (**E**) The relationship between half-maximum concentration and MI. The colored circles in the left subpanel indicate measurements from the preparations in panels **A**-**B**.

Our model predicts that ID responses reflect input from higher affinity ORNs due to receptor saturation, and that differences in the balance of excitation and inhibition should yield differences in the degree of non-monotonicity (Figure 1D-F). We quantified the heterogeneity of ID responses using a monotonicity index (MI) which assigns monotonic glomeruli (i.e., I) a value of 0, while non-monotonic glomeruli (e.g., ID) are assigned increasingly negative values depending on the magnitude of the decrease (Escabí et al., 2007; Higgins et al., 2008). Concentration-response relationships from simultaneously imaged glomeruli in two different preparations illustrate different I and ID responses (Figure 6A-B). We quantified the MI for all responsive glomerulus-odor pairings except for exclusively suppressed glomeruli (D) because they are assigned a strongly negative MI value (Figure 6C). Glomeruli binned into different MI ranges illustrate the heterogeneity of ID responses across the MTC population (Figure 6C). Within the population of non-suppressed MTC glomeruli, 53% exhibited some degree of non-monotonicity (Figure 6D).

We quantified the sensitivity of each MTC glomerulus with piecewise linear interpolation of each concentration-response relationship which was used to quantify the concentration evoking the half maximum response (Figure 6A-B, dashed vertical lines). In this analysis, glomeruli in which the response to the lowest concentration was greater than the half-maximum were assigned a value of 0.05% (e.g., Figure 6B, e.g., roi 9). ID glomeruli with more negative MI values tended to have lower half-maximum values in our exemplar preparations (Figure 6A-B, vertical lines), and across the population (Figure 6E, left panel, 324 glomerulus-odor pairings across 17 preparation-odor pairings). This relationship was significantly correlated (r = 0.48, p = 2.82e-20), and the half-maximum value of I glomeruli was significantly higher than ID glomeruli (Figure 6E, right panel, *p <= 0.00046 for all comparisons with MI = 0*). Thus, MTC glomeruli with ID responses tend to reach their half maximum response at lower concentrations than those exhibiting monotonically increasing relationships with odor concentration.

### Response categories are glomerulus and odor-specific.

**Figure 7:**
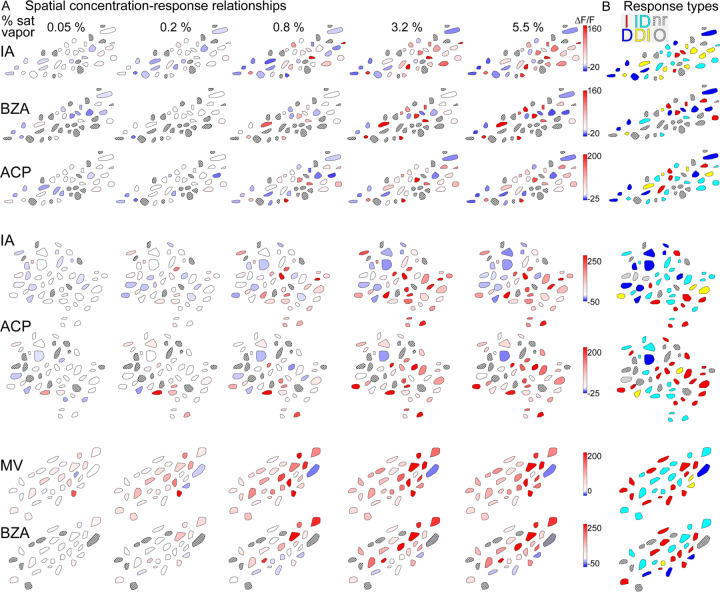
(**A**) Maps of glomerular odor responses (∆F/F) in three different preparations in response todifferent odors. The color scale is fixed across all concentrations for each preparation-odor pairing. Glomeruli that do not respond to any concentration are indicated with a hatched overlay. (**B**) Maps of glomerular response categories: I, Increasing, D, Decreasing; DI, Decreasing then increasing; ID, Increasing then decreasing, nr, Non-responsive; O, Other type not clearly associated with any category. IA, isoamyl acetate; BZA, benzaldehyde; ACP, acetophenone; MV, methyl valerate.

Our model indicates that different linear and non-linear MTC concentration-response relationships are due to the specific combination of the Hill coefficient and half-activation value of the corresponding ORNs (Figure 1E). One prediction of this is that the response category assigned to each glomerulus should be odor-specific. We tested this in a subset of preparations in which responses were measured to at least 2 different odors. Activity maps (ΔF/F) illustrate that response patterns were concentration-dependent and odor-specific (Figure 7A). The different response categories of each glomerulus were identified using the previously described criteria and were mapped using different colors. For each preparation, the same glomerulus could exhibit the same kind of response, or entirely different categorical responses (Figure 7B). There was no evident consistent clustering of glomeruli with the same response type.

## Discussion

We generated a model of the OB input-output transformation that yielded predictions about how the MTC output of individual glomeruli is shaped by intraglomerular and interglomerular mechanisms. Different ORNs were transformed into different output response types that were determined by the Hill coefficient and half-activation value. Notably, our model predicted the presence of ID responses, which depended on lateral inhibition, were heterogeneous, and originated from higher affinity ORN input (Figure 1). *In vivo* measurements from MTC glomeruli revealed that higher concentrations activated increasing numbers of glomeruli, which exhibited a range of different concentration-response categories that were consistent with the model predictions (Figures 2-4). Higher concentrations increased the overall level of mean excitation, which was significantly correlated with the magnitude of suppression present in other glomeruli in the same field of view (Figures 4-5). ID responses were common and heterogeneous in their degree of non-monotonicity and were more sensitive than glomeruli exhibiting exclusively I relationships (Figure 6). The modeling and experimental results indicate that MTC glomerular output is a complex mix of the overall balance of excitation and inhibition innervating that glomerulus.

### Rationale for our modeling choices.

Our mathematical model incorporates intraglomerular and interglomerular processes with strong theoretical and experimental support. Intraglomerular feed-forward inhibition has been proposed as a mechanism for non-topographic contrast enhancement and predicts that inhibition from sensitive interneurons will suppress weak (i.e., presumably non-specific) excitatory ORN input, while stronger inputs (presumably more specific for that ORN) will overcome the feed-forward inhibition (Cleland and Sethupathy, 2006; Cleland, 2010). Experimental support for the existence of this mechanism has been shown anatomically and functionally *in vitro* and *in vivo* (Pinching and Powell, 1971; White, 1972; Gire and Schoppa, 2009; Shao et al., 2009; Economo et al., 2016).

Interglomerular lateral presynaptic inhibition is thought to implement a normalization mechanism that can make the glomerular activation pattern more stable across different concentrations (Cleland and Sethupathy, 2006; Cleland et al., 2007; Cleland, 2010; Cleland et al., 2011). Support for the existence of this mechanism has been shown anatomically and functionally as well (Aungst et al., 2003; Olsen and Wilson, 2008; Cleland, 2010; Olsen et al., 2010; Zhu et al., 2013; Banerjee et al., 2015; Storace and Cohen, 2017). The extent and structure of interglomerular inhibitory coupling is unclear (Banerjee et al., 2015; Economo et al., 2016; Zavitz et al., 2020). Although we find that the general categories of response types remain even when the normalization was performed using a selective subset of ORNs, future studies are needed that systematically compare how different normalization methods match empirical data.

### Comparison with previous studies and methodological considerations.

The presence of both linear and non-linear response types is in contrast with two prior studies reporting that MTC glomeruli exhibit exclusively monotonic concentration-response relationships (Fletcher et al., 2009; Storace and Cohen, 2017). Because linear and non-linear response types were often present in the same imaging field of view, the nonlinear responses cannot be explained as technical limitations associated with our olfactometer, variations in the animal’s respiration, or odor chemistry. In addition, as the model shows, nonlinear glomerular responses would be expected given what is known about local and interglomerular inhibition (Figure 1) (Cleland et al., 2011). We propose that these differences primarily reflect the use of epifluorescence versus 2-photon microscopy, which rejects significantly more out-of-focus fluorescence. Epifluorescence measurements from a region of interest will likely reflect a complicated average of the neighboring pixels, which Figure 7 illustrates can include a combination of I, D, DI and ID glomeruli (Orbach and Cohen, 1983). This is also supported by the observation that MTC glomeruli exhibit less steep concentration-response relationships than their corresponding olfactory receptor neuron input when using epifluorescence imaging (Storace and Cohen, 2017; Storace et al., 2019; Leong and Storace, 2024).

Current models of how inhibitory circuits are shaped by excitation propose that inhibition either increases broadly across the glomerular population in a manner that scales with the magnitude of excitatory input, or increases selectively (Cleland et al., 2007; Banerjee et al., 2015; Economo et al., 2016; Zavitz et al., 2020). ID responses have been rarely described at the level of the input to the olfactory bulb (Wachowiak and Cohen, 2001; Bozza et al., 2004; Carey et al., 2009; Lecoq et al., 2009; Storace and Cohen, 2017; Inagaki et al., 2020; Zak et al., 2020; Martelli and Storace, 2021; Platisa et al., 2022). Our theoretical and experimental results suggest that ID glomeruli reflect processing that occurs within the OB as higher affinity ORN input saturates and is weakened due to rising inhibition (Figures 1 and 6). The result that higher levels of suppression are significantly correlated with increasing excitation is consistent with studies indicating that the responses of interneurons that mediate lateral connectivity exhibit activity that scales with increasing odor concentrations (Banerjee et al., 2015; Storace et al., 2019).

However, another study reported that while the amount of excitation present across MTC glomeruli strongly predicted the proportion of suppressed glomeruli, it was poorly predictive of the amount of suppression (Economo et al., 2016). We propose that a few methodological differences can explain the different conclusions. Our study sampled each glomerular field of view across a large range of odor concentrations, which increased the likelihood that we would observe glomeruli exhibiting this relationship. Additionally, glomeruli that were suppressed at the highest concentration were selected for analysis and their responses to each concentration were analyzed even if they were non-responsive at lower concentrations. This facilitated the identification of glomeruli with graded transitions from non-responsive to suppressed. Finally, we excluded glomeruli that we categorized as DI, which typically responded with suppression to lower concentrations, and transitioned to excitation at higher concentrations. Including DI glomeruli would weaken the relationship between excitation and suppression.

Our finding that more than half of the MTC glomeruli exhibit monotonic concentration-response relationships suggests it is ubiquitous. We also found that most MTC glomeruli exhibiting monotonic concentration-response relationships were well fit by the Hill equation and have Hill coefficients consistent with previous ORN measurements (Wachowiak and Cohen, 2001; Storace and Cohen, 2017; Zak et al., 2020; Platisa et al., 2022). This does not mean that these MTC glomeruli are not affected by inhibitory input. In fact, in the model all glomeruli are subject to both local and lateral inhibition, but in many cases the concentration-response function is monotonically increasing (Figure 1D, I). Future studies that simultaneously compare the ORN and MTC signals from the same glomerulus across concentrations are required to resolve the question of how each individual glomerulus is distinctly impacted by OB processing.

Non-monotonic intensity relationships (“ID” in our study) have been described in other sensory systems as a mechanism by which the brain can encode absolute or relative intensity differences (Sutter and Loftus, 2003; Wehr and Zador, 2003; Polley et al., 2007; Higgins et al., 2010). In the auditory cortex, non-monotonic neurons exhibit unique characteristics including being particularly sensitive to behavioral training and exhibiting unique adaptive properties (Polley et al., 2004; Polley et al., 2006; Watkins and Barbour, 2008). Although our model indicates that these glomeruli are a natural consequence of the interplay between intra- and interglomerular inhibition, it remains to be tested whether these strongly suppressed glomeruli are functionally unique.

### Future studies and conclusions.

It is necessary to know the concentration-response relationships of the input to each glomerulus to further understand the transformation occurring within each glomerulus. Knowing the input-output relationship will allow for additional tests of our proposed model, including a more precise test of the relationship between affinity and ID responses (Figure 1). Importantly, the model predicted that DI responses required PG interneurons, while ID responses required lateral inhibition. Future studies are needed to test this hypothesis by measuring concentration-response relationships before and after selectively manipulating the different cell types involved in these two different computations (Banerjee et al., 2015). To understand the relationship between excitation, suppression and how they are related spatially, experiments are required that incorporate more precise control over the input activation pattern to the brain (Smear et al., 2013; Braubach et al., 2018; Burton et al., 2022).

There are several extensions to the mathematical model presented here that can be implemented to embellish the rich dynamics of ORNs and the OB. Our model here is coarse-grained and assumes *ad hoc* the equilibrium relationship between ORN activity and odorant concentration whilst forgoing temporal dynamics that result in such equilibria. This was intentional, since the simplicity of the model illustrates well that no features other than local and lateral inhibition are needed to replicate the types of responses that we see in our calcium imaging data. Temporal dynamics of glomerular responses to an odor could be incorporated into the model by using time-dependent differential equations, both at the level of ORNs and MTCs.

The model can also be further developed to consider the impact of different interglomerular inhibitory network structures (Zavitz et al., 2020). We performed some preliminary work in this direction, replacing all-to-all coupling with random sampling of the glomeruli providing the lateral inhibition. However, much more can be done to test the effects of different inhibitory network structures. Spatial effects may be heterogeneous, for example, and there could be spatial or functional clustering, so that the lateral inhibition targeted to a glomerulus could be preferentially from nearby glomeruli, or from glomeruli responsive to very distinct odors. The primary conclusion from this study is that nonlinear concentration-responses to MTC glomeruli are quite common, and the model shows that these are in fact expected given the ways that local and lateral inhibition can shape MTC activity.

## Figures and Tables

**Table 1:** Parameter values used in model simulations

Symbol	Value(s)	Description
[odorant]	[0,1]	concentration of input stimuli
* v *	2	Maximum ORN value
* n *	random(1,4)	ORN Hill coefficient
* κ *	random(0,2)	ORN half-activation value
* β *	0.6	Maximum PG value
* $\kappa_{p}$ *	0.5	PG half-activation value
* $\kappa_{m}$ *	1	MTI half-activation value

## Key Points

We developed a mathematical model of the olfactory bulb that incorporates feed-forward and lateral inhibition that transformed monotonic concentration-response inputs into linear and non-linear outputs that were dependent on the input Hill coefficient, half-activation value and rising inhibition.

*In vivo* 2-photon imaging from mitral/tufted (MTC) glomeruli in awake mice revealed similar concentration-response profiles that were odor-specific, and rising levels of suppression that were significantly correlated with excitation.

Non-linear responses in the OB output are common and can be explained by a combination of intra- and interglomerular inhibition.

## References

[R1] Araneda RC, Kini AD, Firestein S (2000) The molecular receptive range of an odorant receptor. Nat Neurosci 3:1248–1255. 11100145 10.1038/81774

[R2] Aungst JL, Heyward PM, Puche AC, Karnup SV, Hayar A, Szabo G, Shipley MT (2003) Centre-surround inhibition among olfactory bulb glomeruli. Nature 426:623–629. 14668854 10.1038/nature02185

[R3] Banerjee A, Marbach F, Anselmi F, Koh MS, Davis MB, Garcia da Silva P, Delevich K, Oyibo HK, Gupta P, Li B, Albeanu DF (2015) An Interglomerular Circuit Gates Glomerular Output and Implements Gain Control in the Mouse Olfactory Bulb. Neuron 87:193–207. 26139373 10.1016/j.neuron.2015.06.019 PMC4633092

[R4] Bozza T, McGann JP, Mombaerts P, Wachowiak M (2004) In vivo imaging of neuronal activity by targeted expression of a genetically encoded probe in the mouse. Neuron 42:9–21. 15066261 10.1016/s0896-6273(04)00144-8

[R5] Braubach O, Tombaz T, Geiller T, Homma R, Bozza T, Cohen LB, Choi Y (2018) Sparsened neuronal activity in an optogenetically activated olfactory glomerulus. Sci Rep 8:14955. 30297851 10.1038/s41598-018-33021-w PMC6175855

[R6] Buck L, Axel R (1991) A novel multigene family may encode odorant receptors: a molecular basis for odor recognition. Cell 65:175–187. 1840504 10.1016/0092-8674(91)90418-x

[R7] Burton SD, Brown A, Eiting TP, Youngstrom IA, Rust TC, Schmuker M, Wachowiak M (2022) Mapping odorant sensitivities reveals a sparse but structured representation of olfactory chemical space by sensory input to the mouse olfactory bulb. Elife 11. 10.7554/eLife.80470 PMC9352350 35861321

[R8] Carandini M (2012) From circuits to behavior: a bridge too far? Nat Neurosci 15:507–509. 22449960 10.1038/nn.3043

[R9] Carandini M, Heeger DJ (2011) Normalization as a canonical neural computation. Nat Rev Neurosci 13:51–62. 22108672 10.1038/nrn3136 PMC3273486

[R10] Carey RM, Verhagen JV, Wesson DW, Pirez N, Wachowiak M (2009) Temporal structure of receptor neuron input to the olfactory bulb imaged in behaving rats. J Neurophysiol 101:1073–1088. 19091924 10.1152/jn.90902.2008 PMC2657066

[R11] Cleland TA (2010) Early transformations in odor representation. Trends Neurosci 33:130–139. 20060600 10.1016/j.tins.2009.12.004 PMC2839009

[R12] Cleland TA, Sethupathy P (2006) Non-topographical contrast enhancement in the olfactory bulb. BMC Neurosci 7:7. 16433921 10.1186/1471-2202-7-7 PMC1368991

[R13] Cleland TA, Johnson BA, Leon M, Linster C (2007) Relational representation in the olfactory system. Proc Natl Acad Sci U S A 104:1953–1958. 17261800 10.1073/pnas.0608564104 PMC1794271

[R14] Cleland TA, Chen SY, Hozer KW, Ukatu HN, Wong KJ, Zheng F (2011) Sequential mechanisms underlying concentration invariance in biological olfaction. Front Neuroeng 4:21. 22287949 10.3389/fneng.2011.00021 PMC3251820

[R15] Daigle TL (2018) A Suite of Transgenic Driver and Reporter Mouse Lines with Enhanced Brain-Cell-Type Targeting and Functionality. Cell 174:465–480 e422. 30007418 10.1016/j.cell.2018.06.035 PMC6086366

[R16] Economo MN, Hansen KR, Wachowiak M (2016) Control of Mitral/Tufted Cell Output by Selective Inhibition among Olfactory Bulb Glomeruli. Neuron 91:397–411. 27346531 10.1016/j.neuron.2016.06.001 PMC6474342

[R17] Escabí MA, Higgins NC, Galaburda AM, Rosen GD, Read HL (2007) Early cortical damage in rat somatosensory cortex alters acoustic feature representation in primary auditory cortex. Neuroscience 150:970–983. 18022327 10.1016/j.neuroscience.2007.07.054

[R18] Fantana AL, Soucy ER, Meister M (2008) Rat olfactory bulb mitral cells receive sparse glomerular inputs. Neuron 59:802–814. 18786363 10.1016/j.neuron.2008.07.039

[R19] Firestein S, Zufall F (1993) Membrane currents and mechanisms of olfactory transduction. Ciba Found Symp 179:115–126; discussion 126–130, 147–119. 8168373 10.1002/9780470514511.ch8

[R20] Fletcher ML, Masurkar AV, Xing J, Imamura F, Xiong W, Nagayama S, Mutoh H, Greer CA, Knopfel T, Chen WR (2009) Optical imaging of postsynaptic odor representation in the glomerular layer of the mouse olfactory bulb. J Neurophysiol 102:817–830. 19474178 10.1152/jn.00020.2009 PMC2724327

[R21] Gire DH, Schoppa NE (2009) Control of on/off glomerular signaling by a local GABAergic microcircuit in the olfactory bulb. J Neurosci 29:13454–13464. 19864558 10.1523/JNEUROSCI.2368-09.2009 PMC2786286

[R22] Higgins NC, Storace DA, Escabi MA, Read HL (2010) Specialization of binaural responses in ventral auditory cortices. J Neurosci 30:14522–14532. 20980610 10.1523/JNEUROSCI.2561-10.2010 PMC3842487

[R23] Higgins NC, Escabí MA, Rosen GD, Galaburda AM, Read HL (2008) Spectral processing deficits in belt auditory cortex following early postnatal lesions of somatosensory cortex. Neuroscience 153:535–549. 18384966 10.1016/j.neuroscience.2008.01.073

[R24] Hu XS, Ikegami K, Vihani A, Zhu KW, Zapata M, de March C, Do M, Vaidya N, Kucera G, Bock C, Jiang Y, Yohda M, Matsunami H (2020) Concentration-dependent recruitment of mammalian odorant receptors. eNeuro. 10.1523/ENEURO.0103-19.2019 PMC7189481 32015097

[R25] Igarashi KM, Ieki N, An M, Yamaguchi Y, Nagayama S, Kobayakawa K, Kobayakawa R, Tanifuji M, Sakano H, Chen WR, Mori K (2012) Parallel mitral and tufted cell pathways route distinct odor information to different targets in the olfactory cortex. J Neurosci 32:7970–7985. 22674272 10.1523/JNEUROSCI.0154-12.2012 PMC3636718

[R26] Inagaki S, Iwata R, Iwamoto M, Imai T (2020) Widespread Inhibition, Antagonism, and Synergy in Mouse Olfactory Sensory Neurons In Vivo. Cell Reports 31. 10.1016/j.celrep.2020.107814 32610120

[R27] Kanter BR, Lykken CM, Moser EI, Moser MB (2022) Neuroscience in the 21st century: circuits, computation, and behaviour. Lancet Neurol 21:19–21. 34942127 10.1016/S1474-4422(21)00427-0

[R28] Langdon C, Genkin M, Engel TA (2023) A unifying perspective on neural manifolds and circuits for cognition. Nat Rev Neurosci 24:363–377. 37055616 10.1038/s41583-023-00693-x PMC11058347

[R29] Lecoq J, Tiret P, Charpak S (2009) Peripheral adaptation codes for high odor concentration in glomeruli. J Neurosci 29:3067–3072. 19279243 10.1523/JNEUROSCI.6187-08.2009 PMC6666444

[R30] Leong LM, Storace DA (2024) Imaging different cell populations in the mouse olfactory bulb using the genetically encoded voltage indicator ArcLight. Neurophotonics 11:033402. 38288247 10.1117/1.NPh.11.3.033402 PMC10823906

[R31] Malnic B, Hirono J, Sato T, Buck LB (1999) Combinatorial receptor codes for odors. Cell 96:713–723. 10089886 10.1016/s0092-8674(00)80581-4

[R32] Martelli C, Storace DA (2021) Stimulus Driven Functional Transformations in the Early Olfactory System. Frontiers in Cellular Neuroscience 15. 10.3389/fncel.2021.684742 PMC8369031 34413724

[R33] McGann JP (2013) Presynaptic inhibition of olfactory sensory neurons: new mechanisms and potential functions. Chem Senses 38:459–474. 23761680 10.1093/chemse/bjt018 PMC3685425

[R34] Mitsui S, Igarashi KM, Mori K, Yoshihara Y (2011) Genetic visualization of the secondary olfactory pathway in Tbx21 transgenic mice. Neural Syst Circuits 1:5. 22330144 10.1186/2042-1001-1-5 PMC3257540

[R35] Nagayama S, Homma R, Imamura F (2014) Neuronal organization of olfactory bulb circuits. Front Neural Circuits 8:98. 25232305 10.3389/fncir.2014.00098 PMC4153298

[R36] Nagayama S, Enerva A, Fletcher ML, Masurkar AV, Igarashi KM, Mori K, Chen WR (2010) Differential axonal projection of mitral and tufted cells in the mouse main olfactory system. Front Neural Circuits 4. 10.3389/fncir.2010.00120 PMC2952457 20941380

[R37] Olsen SR, Wilson RI (2008) Lateral presynaptic inhibition mediates gain control in an olfactory circuit. Nature 452:956–960. 18344978 10.1038/nature06864 PMC2824883

[R38] Olsen SR, Bhandawat V, Wilson RI (2010) Divisive normalization in olfactory population codes. Neuron 66:287–299. 20435004 10.1016/j.neuron.2010.04.009 PMC2866644

[R39] Orbach HS, Cohen LB (1983) Optical monitoring of activity from many areas of the in vitro and in vivo salamander olfactory bulb: a new method for studying functional organization in the vertebrate central nervous system. J Neurosci 3:2251–2262. 6631479 10.1523/JNEUROSCI.03-11-02251.1983 PMC6564639

[R40] Parrish-Aungst S, Shipley MT, Erdelyi F, Szabo G, Puche AC (2007) Quantitative analysis of neuronal diversity in the mouse olfactory bulb. J Comp Neurol 501:825–836. 17311323 10.1002/cne.21205

[R41] Pinching AJ, Powell TP (1971) The neuropil of the glomeruli of the olfactory bulb. J Cell Sci 9:347–377. 4108057 10.1242/jcs.9.2.347

[R42] Platisa J, Zeng H, Madisen L, Cohen LB, Pieribone VA, Storace DA (2022) Voltage imaging in the olfactory bulb using transgenic mouse lines expressing the genetically encoded voltage indicator ArcLight. Sci Rep 12:1875. 35115567 10.1038/s41598-021-04482-3 PMC8813909

[R43] Polley DB, Steinberg EE, Merzenich MM (2006) Perceptual learning directs auditory cortical map reorganization through top-down influences. J Neurosci 26:4970–4982. 16672673 10.1523/JNEUROSCI.3771-05.2006 PMC6674159

[R44] Polley DB, Read HL, Storace DA, Merzenich MM (2007) Multiparametric auditory receptive field organization across five cortical fields in the albino rat. J Neurophysiol 97:3621–3638. 17376842 10.1152/jn.01298.2006

[R45] Polley DB, Heiser MA, Blake DT, Schreiner CE, Merzenich MM (2004) Associative learning shapes the neural code for stimulus magnitude in primary auditory cortex. Proc Natl Acad Sci U S A 101:16351–16356. 15534214 10.1073/pnas.0407586101 PMC528983

[R46] Reisert J, Matthews HR (1999) Adaptation of the odour-induced response in frog olfactory receptor cells. J Physiol 519 Pt 3:801–813. 10457092 10.1111/j.1469-7793.1999.0801n.x PMC2269541

[R47] Shao Z, Puche AC, Kiyokage E, Szabo G, Shipley MT (2009) Two GABAergic intraglomerular circuits differentially regulate tonic and phasic presynaptic inhibition of olfactory nerve terminals. J Neurophysiol 101:1988–2001. 19225171 10.1152/jn.91116.2008 PMC2695638

[R48] Smear M, Resulaj A, Zhang J, Bozza T, Rinberg D (2013) Multiple perceptible signals from a single olfactory glomerulus. Nat Neurosci 16:1687–1691. 24056698 10.1038/nn.3519 PMC13445371

[R49] Storace DA, Cohen LB (2017) Measuring the olfactory bulb input-output transformation reveals a contribution to the perception of odorant concentration invariance. Nat Commun 8:81. 28724907 10.1038/s41467-017-00036-2 PMC5517565

[R50] Storace DA, Cohen LB (2021) The mammalian olfactory bulb contributes to the adaptation of odor responses: a second perceptual computation carried out by the bulb. eNeuro. 10.1523/ENEURO.0322-21.2021 PMC8474650 34380657

[R51] Storace DA, Cohen LB, Choi Y (2019) Using Genetically Encoded Voltage Indicators (GEVIs) to Study the Input-Output Transformation of the Mammalian Olfactory Bulb. Front Cell Neurosci 13:342. 31417362 10.3389/fncel.2019.00342 PMC6684792

[R52] Subramanian N, Leong LM, Salemi Mokri Boukani P, Storace DA (2025) Recent odor experience selectively modulates olfactory sensitivity across the glomerular output in the mouse olfactory bulb. Chem Senses 50. 10.1093/chemse/bjae045 PMC11753175 39786438

[R53] Sutter ML, Loftus WC (2003) Excitatory and inhibitory intensity tuning in auditory cortex: evidence for multiple inhibitory mechanisms. J Neurophysiol 90:2629–2647. 12801894 10.1152/jn.00722.2002

[R54] Verhagen JV, Wesson DW, Netoff TI, White JA, Wachowiak M (2007) Sniffing controls an adaptive filter of sensory input to the olfactory bulb. Nat Neurosci 10:631–639. 17450136 10.1038/nn1892

[R55] Wachowiak M, Cohen LB (2001) Representation of odorants by receptor neuron input to the mouse olfactory bulb. Neuron 32:723–735. 11719211 10.1016/s0896-6273(01)00506-2

[R56] Watkins PV, Barbour DL (2008) Specialized neuronal adaptation for preserving input sensitivity. Nat Neurosci 11:1259–1261. 18820690 10.1038/nn.2201

[R57] Wehr M, Zador AM (2003) Balanced inhibition underlies tuning and sharpens spike timing in auditory cortex. Nature 426:442–446. 14647382 10.1038/nature02116

[R58] White EL (1972) Synaptic organization in the olfactory glomerulus of the mouse. Brain Res 37:69–80. 4334289 10.1016/0006-8993(72)90346-0

[R59] Xu L, Li W, Voleti V, Zou DJ, Hillman EMC, Firestein S (2020) Widespread receptor-driven modulation in peripheral olfactory coding. Science 368. 10.1126/science.aaz5390 PMC7443284 32273438

[R60] Zak JD, Reddy G, Vergassola M, Murthy VN (2020) Antagonistic odor interactions in olfactory sensory neurons are widespread in freely breathing mice. Nat Commun 11:3350. 32620767 10.1038/s41467-020-17124-5 PMC7335155

[R61] Zavitz D, Youngstrom IA, Borisyuk A, Wachowiak M (2020) Effect of Interglomerular Inhibitory Networks on Olfactory Bulb Odor Representations. J Neurosci 40:5954–5969. 32561671 10.1523/JNEUROSCI.0233-20.2020 PMC7392506

[R62] Zhu P, Frank T, Friedrich RW (2013) Equalization of odor representations by a network of electrically coupled inhibitory interneurons. Nat Neurosci 16:1678–1686. 24077563 10.1038/nn.3528

